# SARS-CoV-2 direct real-time polymerase chain reaction testing in laboratories with shortage challenges

**DOI:** 10.2217/fvl-2020-0187

**Published:** 2021-02-03

**Authors:** Taghreed Alaifan, Asmaa Altamimi, Dalia Obeid, Turki Alshehri, Shaihana Almatrrouk, Ahmed Albarrag

**Affiliations:** 1 ^1^Saudi Center for Disease Control & Prevention, Public Health Laboratory, Riyadh, Saudi Arabia; 2 ^2^Department of Pathology, Medical Microbiology, College of Medicine, King Saud University, Riyadh, Saudi Arabia

**Keywords:** COVID-19, direct-RT-PCR, methodology, pandemic, rapid testing, RNA extraction, RT-PCR validation, SARS-CoV-2, surveillance in developing countries, viral-detection

## Abstract

**Aim:** In our study, we propose the use of direct real-time polymerase chain reaction (RT-PCR), this test does not require extraction or a preheating step, which saves a lot of cost, labor, processing time and provides a solution for supply shortage. **Materials & methods:** We assayed 185 nasopharyngeal samples stored in viral transport media. The indirect method was done with RNA extraction step, and the direct RT-PCR was done without an extraction step, both assays were evaluated on a commercially validated severe acute respiratory syndrome coronavirus 2 (SARS-CoV-2) kit targeting *E* gene. **Results:** Our assay showed a sensitivity of 79%, a specificity of 100% and the agreement between methods was 72%. **Conclusion:** Overall, this simple direct RT-PCR approach can be utilized as a qualitative diagnostic tool for emergency SARS-CoV-2 surveillance in countries with limited resources and may help laboratories to continue testing and at greater frequency despite supply shortages, although with delay in cycle threshold value in comparison with indirect RT-PCR.

The ongoing coronavirus pandemic has placed constraints on health system capabilities worldwide. As of January 2021, around 83 million people were infected with the virus, and it caused more than 1.8 million deaths globally [1]. This pandemic is caused by the SARS-CoV-2 virus, the virus shows high genetic resemblances with RaTG13 and Pangolin coronaviruses viruses [2,3]. In 2009, the development of an effective vaccine during the influenza strain A(H1N1) pandemic was very useful in stopping the spread of the virus, however, some of the SARS-CoV-2 vaccine candidates are still at the testing phase and many countries may struggle with supplies and cost to cover the population [4,5]. Additionally, healthcare systems worldwide are being used at full capacity and there are still fears with the rapid incline of cases, the current systems will fail.

One of the most important aspects of rapid response is developing prompt testing. The current gold standard of testing relies on qualitative reverse real-time polymerase chain reaction (RT-PCR) from nasal or pharyngeal swabs stored in a viral transport medium (VTM) [6,7]. The process of detecting the virus starts with RNA extraction, then either cDNA synthesis or directly RT-PCR depending on the kit available. The turnaround time (TAT), which is the time interval between receiving the sample in the laboratory and result reporting, for a common RT-PCR test is around 24–48 h, however, with the pandemic reaching higher numbers, the speed of testing may be delayed and that affect outbreak control [8]. In fact, the increased demand for COVID-19 testing in public health laboratories can affect the TAT for other viruses testing such as measles in South Sudan [9]. The encumbrance of late (TAT) will not only have affects in clinical settings, but it will also affect the economy significantly.

Therefore, there are many efforts globally to develop rapid testing, however as many of them are based on serology, lots of them are not sensitive enough in clinical settings [10]. Consequently, as an alternative to shortening RT-PCR time has been proposed the use of the loop-mediated isothermal amplification method [11]. Thus, focusing on improving the workflow of RT-PCR is very essential as it ensures high sensitivity and specificity in clinical settings.

One of the most successful attempts in improving diagnostic testing was reported in South Korea. South Korea increased its testing capacity by recruiting domestic companies to provide testing kits, which have shortened the supply leading time drastically [12]. This approach is applicable in countries with biomedical resources and specialized factories, however, many countries either don't have these advancements in the biomedical field or cannot afford the cost.

Direct PCR amplification from samples is a technique that could be useful in the current phase of the pandemic. Direct amplification can tremendously shorten (TAT) time and reduce testing costs by eliminating the extraction phase of the samples handling workflow. Moreover, this kind of method reduces the sample handling time which will decrease the probability of contamination across the samples. This method has shown previously promising results in bacterial culture, human cells and plants [13,14]. Considering the current pandemic, the use of direct RT-PCR offers an interesting solution for laboratories with limited supplies and reagents.

In our current study, we provide an alternative method that provides diagnostic results without the need for the RNA extraction step or any additives. This protocol is useful in laboratories with limited resources and infrequent supplies. The direct RT-PCR uses samples stored in VTM directly into the RT-PCR reaction. In our study we evaluated our assay, by comparing it to the nationally validated method in Saudi Arabia, to determine its sensitivity and specificity. Overall, we aim to deliver a cost-effective method for laboratories with supply challenges and that are overwhelmed with mass population samples.

## Methods

### Sample selection

Specimens were selected from the diagnostic laboratories at the Saudi Center for Diseases Control and Prevention (SCDC). 185 clinical nasopharyngeal swabs (124 COVID-19 positives and 64 negatives) stored at -80°C in 2 ml of VTM (Vicell, Spain) were tested by both the indirect RT-PCR method and the direct RT-PCR method. Samples included in our study were collected from regional laboratories across Saudi Arabia. To draw a fair evaluation for our methodology, we choose samples with variation in their cycle threshold (Ct) values.

### Pilot experiment

Initially, we aimed to evaluate the appropriate sample volume input in the RT-PCR and the effect of the heating step on the direct technique. Nine samples with Ct values ranged from 18 to 28 selected randomly. A total of 3, 5 and 10 μl of each sample were added respectively to 10 μl of master mix. Also, 40 previously positive samples with different Ct values ranging from 18 to 36 were selected randomly. Aliquots of 100 μl from each VTM were heated in a water bath at 60°C for 15 min. Then a 3 μl of heat-treated samples and a 3 μl without heating were subjected directly to RT-PCR.

### Standard method (indirect) reverse RT-PCR method

For the indirect RT-PCR test, RNA was extracted on the 96 MagNA Pure instrument (Roche, Mannheim, Germany) using the MagNA Pure 96 DNA and Viral NA Small volume isolation Kit. The extraction protocol was done following the manufacture protocol using 200-μl sample input and producing 50-μl of RNA output. The RNA was then used in one-step RT-PCR, using LightMix^®^ Modular RNA master mix (Roche, Cat. No. 06754155001) with *E* gene LightMix^®^ as a target (Roche, Cat:53077696) to detect SARS-CoV-2 virus according to the manufacturer instructions. Briefly, a final reaction volume of 15 μl containing; 5.5 μl of DNAse/RNAse free Water, 0.5 μl of Reagent mix including probes and primers, 4.0 μl of Roche Master Mix, 0.1 μl of RT enzymes and 10 μl of RNA template (samples or control). The RT-PCR reaction was performed on the Light Cycler 480 II instrument (Roche) following the recommended cycling conditions: reverse transcription at 55°C for 5 min and denatured at 95°C for 5 min, followed by 45 cycles of PCR at 95°C for 5 s, 60°C for 15 s and 72°C for 15 s, and then cooled at for 40°C for 30 s. Samples and control templates (positive and negative) were run in triplicate wells, the Ct value reported was the average of the three wells. A Ct value above 40 was considered a negative result.

### Direct RT-PCR

For our developed protocol, samples that were stored in VTM were vortexed for a minute and 3 μl were then directly used into the RT-PCR LightMix^®^ reaction at the same conditions stated in the indirect RT-PCR method using the LightMix^®^

gene assay.

### Data analysis

For statistical analysis, data were collected and analyzed using SAS software, version 9.4 and Prism, Version 8.4. Sensitivity and specificity statistical measures were used to compare the indirect RT-PCR results with the direct RT-PCR assay. Tables for the qualitative results were analyzed by agreement confidence intervals, McNamara's test and T Paired test. Boxplots and correlation graphs were used to show the distribution of Ct values between these two methods. All p-values reported are two-sided and were considered to be statistically significant at alpha <0.05.

## Results

### Qualitative analysis

The results of the direct RT-PCR compared with the indirect RT-PCR method as a qualitative test is summarized in Table 1. Overall the agreement between the direct method and the indirect method was 72% (p < 0.0001), which is considered a moderate-high agreement. The developed direct RT-PCR method reported a sensitivity of 79.34% and specificity of 100%, furthermore, the probability of the negative likelihood was estimated to be 0.20.

**Table 1.** The qualitative results of the direct real-time polymerase chain reaction and the indirect real-time polymerase chain reaction by agreement statistics.

Direct RT-PCR	Indirect RT-PCR		Total
	Positive (n Sample)	Negative (n Sample)	
Positive	96	0	96
Negative	25	64	86
Total	121	64	185
Concordance statistics, (95% CI)			
Agreement coefficient		0.72 (0.62–0.81)	
Sensitivity		79.34% (71.0–86.16%)	
Specificity		100% (94.1–100.0%)	
McNemar's test (p-value)		23.04 (p < 0.001)	

RT-PCR: Real-time polymerase chain reaction.

### Quantitative analysis on cycle threshold

**Figure 1.**
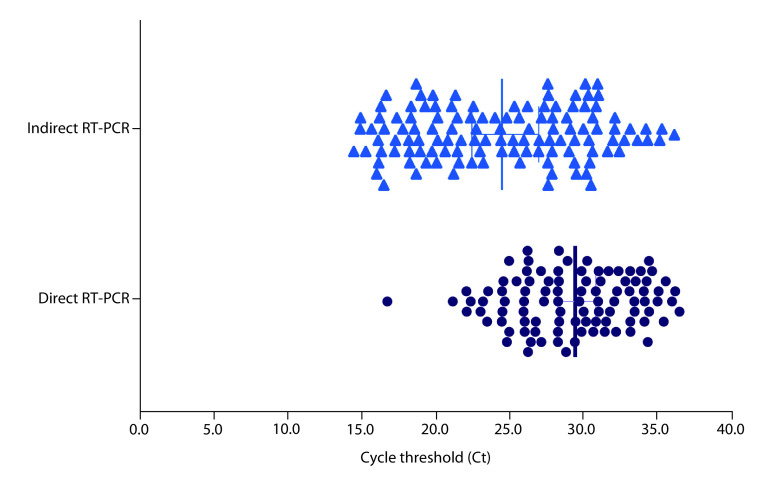
Distribution of the cycle threshold values obtained by the indirect real-time polymerase chain reaction method and the direct real-time polymerase chain reaction method. The mean cycle threshold value of the direct method was 29.64, while the indirect real-time polymerase chain reaction was 24.9.

In the case of the Ct obtained by both methods, quantitative analysis showed late Ct values detected in the direct RT-PCR method compared with the indirect RT-PCR method. The Ct values distribution by methods of detection are shown in Figure 1, overall lower Ct values were shown in the indirect RT-PCR, which is expected, and the change between the methods was significant (t = 5.93; p < 0.0001). The median Ct value reported in the indirect RT-PCR method was 25 and the median Ct value for the direct RT-PCR method was around 30. The mean of the change obtained in the form of Ct values was 5, which is considered high with 95% confidence intervals of (3.4–6.74).

**Figure 2.**
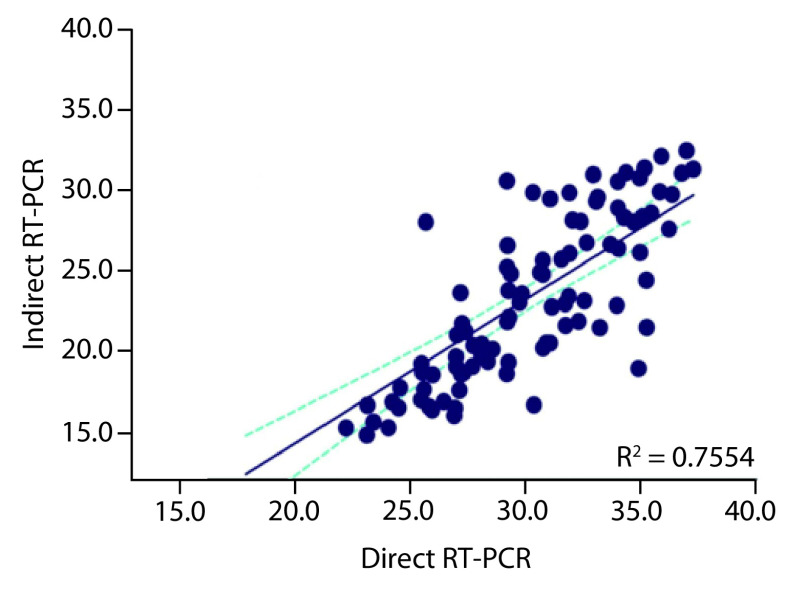
Correlation between cycle threshold values by the indirect real-time polymerase chain reaction method and the direct real-time polymerase chain reaction method obtained by the Lightcycler 480 II instrument. The correlation coefficient of the analysis is R = 0.755 and has a p-value < 0.0001.

The levels of the Ct values by classification show a lower agreement as shown in Table 2. The correlation for the cycle threshold for the samples tested by both methods is shown in Figure 2. The correlation between both methods by Ct values shows a moderate correlation with R^2^ = 0.755 (p < 0.0001). This correlation was shown to be the strongest for low Ct value positive samples.

**Table 2.** The results of the cycle threshold levels obtained by the direct real-time polymerase chain reaction and the indirect real-time polymerase chain reaction by cycle threshold levels and agreement statistics.

Indirect RT-PCR	(n sample)
High (Ct < 25)	61
Intermediate (Ct 25–33)	51
Low (Ct > 33)	9
Negative	64
Total	185
**Direct RT-PCR**	**(N sample)**
High (Ct < 25)	9
Intermediate (Ct 25–33)	62
Low (Ct > 33)	25
Negative	89
Total	185
**Weighted Kappa (Agreement)**	0.23 (-0.51–0.96)

Ct: Cycle threshold; RT-PCR: Real-time polymerase chain reaction.

For the pilot experiment, adding 3 μl of nine samples showed a better S-shape and steep-slope amplification curve, which are indications of a healthy reaction. The presence of sample inhibitors impedes the RT-PCR reaction and interferes with efficiency, therefore, results in a lower curve slope [15], and Ct value although the difference in mean Ct value reported was not significant (Supplementary Materials 1.1). For the heating procedure, 17 samples out of 40 COVID-19 samples showed no significant difference either on Ct value or amplification curve shape. A total 23 sample results showed no amplification curve since their Ct values were >33, therefore we proceeded without the heating step (Supplementary Materials 1.2).

## Discussion

Overall, our study indicates that the use of direct RT-PCR provides concordance testing results compared with the indirect RT-PCR method. The developed detection assay had a 79% sensitivity and 100% specificity. These results indicate that direct RT-PCR can be a reliable method in difficult times when the extraction step is not applicable. However, the test showed high false-negative rates in samples with late Ct values. Lower Ct values were reported in the indirect method, which is expected, and the change in Ct values was significant between these two methods (t = 5.93; p < 0.0001). The median Ct value reported by the indirect method was 25 and the median Ct value for the direct method was 30. The Ct values distribution by the two methods shows that in the early Ct value positive samples high agreement was detected, however in samples with late Ct most of the results were negative. Furthermore, in positive samples, a moderate correlation was observed which indicates that although Ct values were significantly different, the two assays were correlated together. The direct RT-PCR method provided good specificity, but its sensitivity is still lacking, however, in times of need, the usage of this test can be useful in terms of TAT and emergency conditions such as lack of supply and pandemic high demand for testing.

Direct RT-PCR was validated in a preprint during the COVID-19 pandemic [16]. Investigators reported a high sensitivity of 93% and used samples without VTM, in our study we got less sensitivity which could indicate a PCR inhibition. Another study from Washington University also used a similar method starting with preheating the samples before the RT-PCR, their assay was able to detect 92% of the positive samples [17]. Both studies reported higher sensitivity results for the indirect method than ours; however, both studies used samples with lower Ct values. In our study, sample Ct values were ranged from 14 to 35 to avoid bias. High CT (>33) samples mostly give negative results with direct RT-PCR, which affects the sensitivity value significantly.

The Ct value gives an estimate of the amount of target nucleic acid and correlates inversely with viral load, a high Ct value indicates low viral load and vice versa [18]. Higher volume inputs did not improve sensitivity, as with direct RT-PCR the higher the VTM volume the higher the chance of introducing inhibitors as found also in another study [19]. Furthermore, the use of commercial VTMs affects SARS-CoV-2 and Influenza PCR detection as shown in one study [20]. In our direct method, we introduced the VTM components directly into the master mix, which may partially inhibit or interfere with the RT-PCR and cause a delay in Ct values especially samples with low viral load (Ct > 33), which have affected our assay sensitivity.

The direct RT-PCR in the application of DNA viral detection has shown promising results in hepatitis B and Mycoplasma organisms [21,22]. Other studies using direct RT-PCR on detecting viral RNA suggested that the use of PCR enhancers could lead to early RNAase release, which compromises the RNA integrity [23]. PCR inhibition is the leading cause of reporting low testing sensitivity in various fluids, in which some are reported as resistant to heat and demand the extraction step with sample dilution to eliminate the inhibition [24,25].

The main advantages of using this assay are: shortening the diagnostic test time from 2.5 to 1.5 h, increasing the cost–effectiveness, minimizing hands-on labor, which decreases the probability of contamination, and coping with extraction reagent supply challenges [26]. The importance of our work rises in the time of limited supplies, however, the direct assay can be improved by using adjusted lysis reagent and validating the different types of VTMs as they play an important role in introducing PCR inhibitors. In another recent study to assess direct RT-PCR, they found that by using specific types of VTM and controlled inactivation of the virus, the direct RT-PCR is a viable option [18]. For future work, we aim to assess the different types of VTMs and lysis buffers to improve the sensitivity of our assay.

The main limitation of our study is shortage of resources, because of the current time it is hard for many laboratories worldwide to research diagnostic testing. Resources and labors were scarce with high priority for diagnostic testing. The majority of the samples received at the public health laboratories were mostly positive, which could potentially bias the results, but we pooled a sufficient number of negative samples to use in our study. Another limitation of our study is that we used a single gene target to detect the virus, which could have played a role in our high false-negative rate.

## Conclusion

We were able to perform and evaluate a direct and simple RT-PCR test that does not require extraction or step during the SARS-CoV-2 pandemic crisis. The direct RT-PCR approach delivered sufficient results with good concordance with the gold indirect test. Direct RT-PCR could be a valuable solution to identify COVID-19 suspects, although with a lower Ct value, in a shorter time. Therefore, the indirect RT-PCR test could be reserved for individuals with an initial negative result. Our finding provides a simple solution to overcome the RNA extraction supply shortage.

**Summary points**

- In our study, we proposed the use of direct real-time polymerase chain reaction (RT-PCR), this test does not require extraction or preheating step, which saves a lot of cost, labor, processing time and provides a solution for supply shortage.
- The direct RT-PCR assay was validated by comparing it to the nationally validated method in Saudi Arabia, to determine its sensitivity and specificity.
- A total of 185 nasopharyngeal samples stored in validate viral transport media were used for the validation. Both of the indirect RT-PCR protocol, which is based on RNA extraction, and the direct RT-PCR were evaluated on commercially validated SARS-CoV-2 kit targeting *E* gene.
- Overall, the agreement between the direct method and the indirect method was 72% (p < 0.0001), which is considered a moderate agreement.
- The developed direct RT-PCR method reported a sensitivity of 79.34% and specificity of 100%.
- The test showed high false-negative rates in samples with late cycle threshold (Ct) values. Lower Ct values were reported in the indirect method, which is expected, and the change in Ct values was significant between these two methods (t = 5.93, p < 0.0001).
- The correlation between both methods by Ct values shows a moderate correlation with R^2^ = 0.755 (p < 0.0001).
- The main limitations of our study were resources-shortage, sample selection and RT-PCR target.
- Future work should be done to improve the assay sensitivity and validate viral transport media that provide the best results, thus limit the effect of PCR inhibitors.
- This direct RT-PCR approach can be utilized as a qualitative diagnostic tool for emergency SARS-CoV-2 surveillance in countries with limited resources.
