# Supplementary material for: SARS-CoV-2 direct real-time polymerase chain reaction testing in laboratories with shortage challenges
Source: Future Virol. 2021 Feb 3:10.2217/fvl-2020-0187. doi: 10.2217/fvl-2020-0187 (PMC7856914; doi:10.2217/fvl-2020-0187)
Supplement: Supplementary file 1 [file supplementary_materials.docx]

**Supplementary materials**

**S1.1) Sample input Pilot experiment**

To evaluate the efficiency of the RT-PCR master mix we used three different volume inputs from the samples to select the optimal volume. We used 3, 5 and 10 microliters of each sample to 10 microliters of E gene LightMix® master mix. An example of the results of our experiment are showing in figure S1.1. Adding 3 microliters of the sample showed a better S-shape amplification curve with no significant difference in Ct value (Figure S1.1). The results of the experiment showed that an input of 3 microliters have shown to be the best, therefore we carried the rest of our experiments using 3 microliters.

**
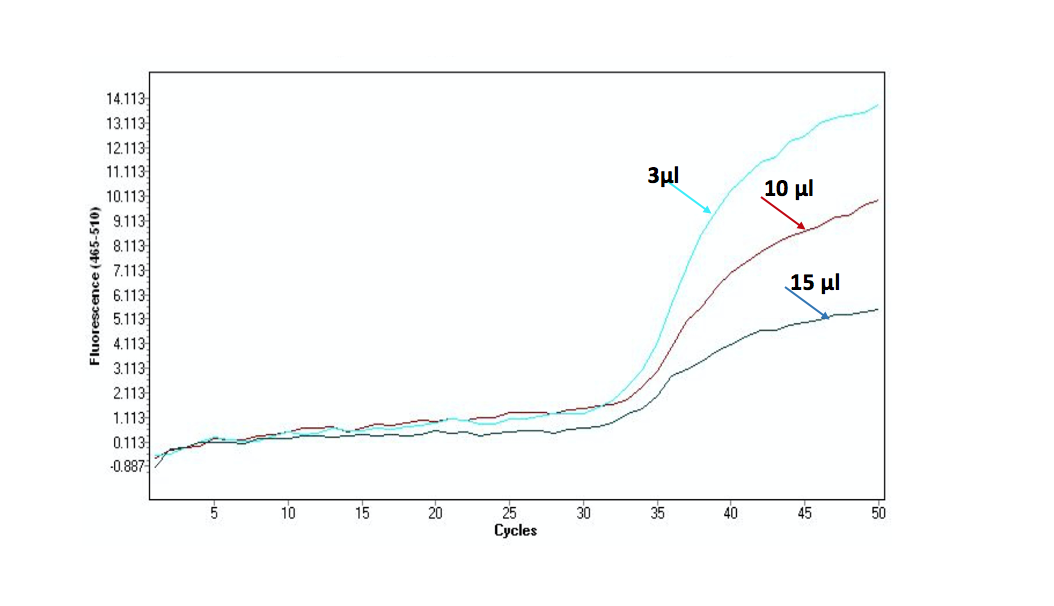
**

**Figure S1.1.** An amplification Plot for direct Real-time PCR Using E gene LightMix® assay by using three different input volumes (3,10,15 microliters) of sample No.2. The plot shows the difference in fluorescence detected (Rn) at each cycle number and amplification curve shape. The light blue color line shows the sample with 3 μl input, the red color line shows the with 5 μlinput, and the dark blue color line shows sample with 10 μl volume input.

**
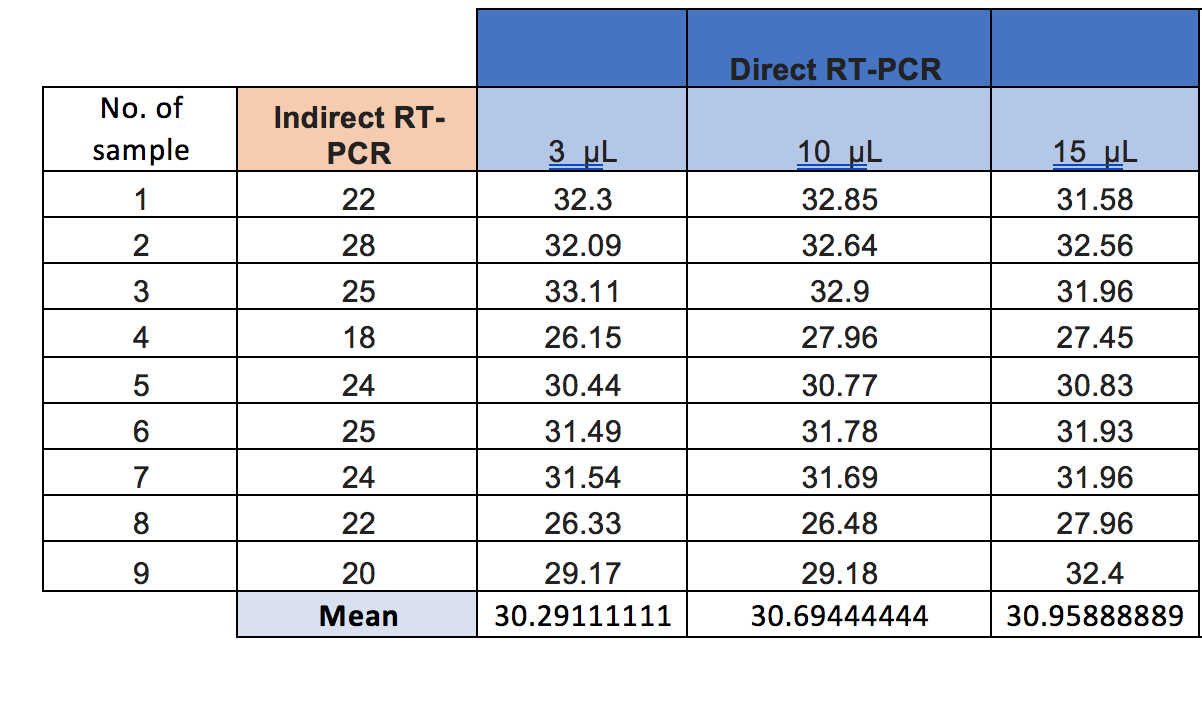
**

**Table S1.1.** The results of the cycle threshold (Ct) levels obtained by the Direct RT-PCR using three different input volumes (3,10,15 microliters) of 9 samples in comparison with Indirect RT-PCR Ct results.

**(S1.2) The results of our pilot experiment for preheating samples prior to RT-PCR**

To evaluate the effect of preheating step on the RT-PCR efficiency we used 40 samples in which we incubated in a water bath at 60 °C for 15 min. Then 3 microliters of each sample have been added, with heating and without heating treatment. The preheating sample treatment showed no difference on Ct values, and nor the amplification curve (Figure S1.2). The results of the experiment showed that preheating treatment for the sample showed no effect on the reported Ct value or the amplification curve therefore we proceeded without the heating step.

**
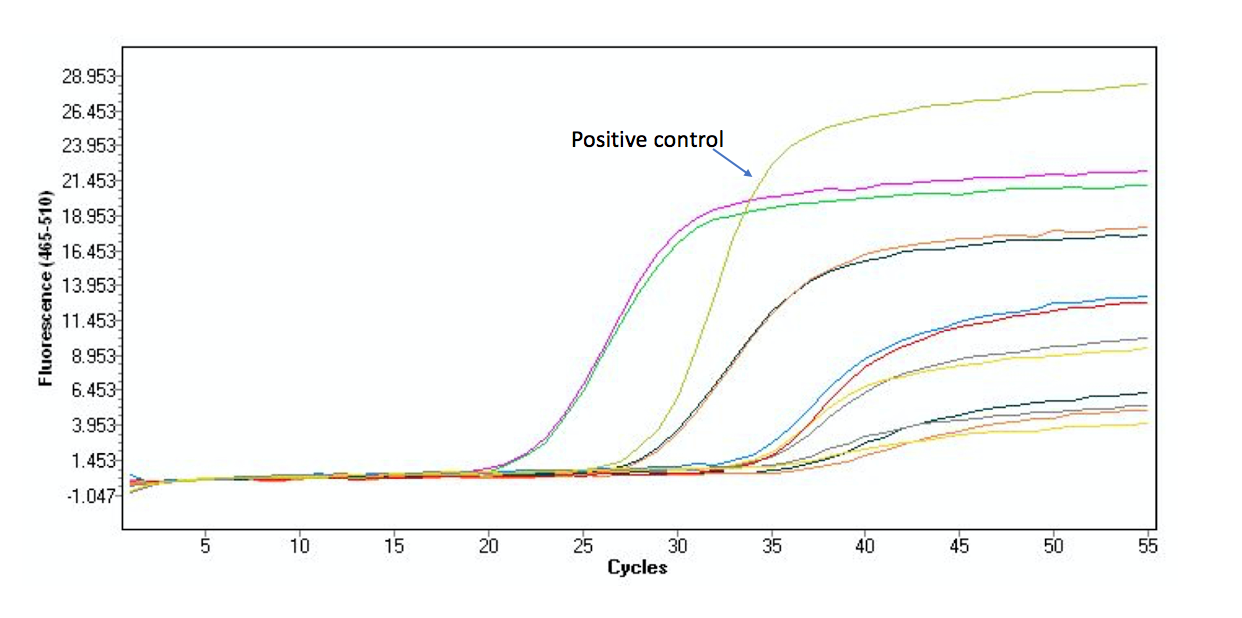
**

**Figure S.1.2.** Amplification Plot for Real-time PCR Using E gene LightMix® assay by preheating and no heating conditions. The plot shows the difference in fluorescence detected (Rn) at each cycle number. No difference between preheated samples and no heated samples was found regarding their CT values and amplification curve.
